# The heterogeneity of cancer stem-like cells at the invasive front

**DOI:** 10.1186/s12935-017-0393-y

**Published:** 2017-02-13

**Authors:** Go J. Yoshida

**Affiliations:** 1 Department of Pathological Cell Biology, Medical Research Institute, Tokyo Medical and Dental University, 1-5-45 Yushima, Bunkyo-ku, Tokyo, 113-8510 Japan; 2 Japan Society for the Promotion of Science, 5-3-1 Kojimachi, Chiyoda-ku, Tokyo, 102-0083 Japan

**Keywords:** Cancer stem-like cells, CD44, c-Myc, ESRP1-CD44v-xCT axis, Heterogeneity, Invasive front, Minimal residual disease, Negative feedback machinery, Reactive oxygen species, Wnt/β-catenin signal

## Abstract

Cancer stem-like cells exhibit the multi-functional roles to survive and persist for a long period in the minimal residual disease after the conventional anti-cancer treatments. Cancer stem-like cells of solid malignant tumors which highly express CD44v8-10, the variant isoform of CD44 generated by alternative splicing, has a resistance to redox stress by the robust production of glutathione mediated by ESRP1-CD44v-xCT (cystine/glutamate antiporter) axis. It has been reported that CD44v and c-Myc tend to show the inversed expression pattern at the invasive front of the aggressive tumors. Given that the accumulation of reactive oxygen species triggers the activation of Wnt/β-catenin signal pathway, it is hypothesized that CD44v causes the negative feedback machinery in the regulation of c-Myc expression via the attenuated ROS-induced Wnt signal pathway. To address the fundamental question whether and how both proliferative and quiescent cancer stem-like cells heterogeneously exist at the invasive/metastatic edge, researchers need to investigate into the E3-ubiquitin ligase activity essential for c-Myc degradation. CSCs heterogeneity at the invasive/metastatic front is expected to demonstrate the dynamic tumor evolution with the selective pressure of anti-cancer treatments. Furthermore, the novel molecular targeting therapeutic strategies would be established to disrupt the finely-regulated c-Myc expression in the heterogeneous CSC population in combination with the typical drug-repositioning with xCT inhibitor.

## Background

Metastatic and relapsed diseases account for more than 90% of cancer deaths. Minimal residual disease (MRD), which is undetectable in the clinical settings, is widely accepted to be responsible for the latent relapse and distant metastasis. The heterogeneous tumor cell population undergoes the selective pressure such as hypoxia, hypo-nutrient conditions, chronic inflammation, and activated innate immune system. The selective pressure of the unfavorable tumor microenvironment is considered to transiently decrease the degree of the intra-tumoral heterogeneity, which is referred to as “bottle-neck effect” in the evolutionary theory [1].

Cancer stem-like cells (CSCs) are defined as the enhanced tumorigenic subpopulation of cancer cells. They exist at the top of the hierarchical tumor cell society composed of CSCs, transit-amplifying cells, and non-CSCs. CSCs exhibit self-renewal potential and can generate numerous progenitor/daughter cells at various degrees of differentiation in much the same way as normal tissue stem cells. Accumulating evidence has strongly suggested that CSCs can persist after currently available anti-cancer medical interventions such as chemotherapy and radiotherapy. Several molecular machineries underlying this therapeutic resistance specific to CSCs have been identified, including the resistance to oxidative stress and chronic inflammation, the maintenance of quiescent status of the cell cycle, a rapid response to DNA damage, and the robust export of cytotoxic agents. Therefore, MRD is enriched in CSCs with high tumorigenic potential and chemo-resistance [2].

Conventional anti-cancer therapies preferentially kill the proliferative non CSCs and proliferative CSCs, which is explains the reason why quiescent CSCs tend to persist in MRD for a long period. Residual dormant CSCs which have survived after the anti-cancer treatment are responsible for the tumor relapse and distant metastasis after their re-entry into the cell cycle. Remarkably, a recent investigation reveals that prostaglandin E_2_ (PGE_2_), the cytokine involved in the chronic inflammation and wound healing, is responsible for the iatrogenic activation of CSCs and the progressive development of chemo-resistance. Cytokeratin14-positive undifferentiated bladder CSCs in the dormant cell cycle G_0_ phase were induced to proliferate and repopulate the MRD on exposure to PGE_2_ which was released from non-CSC cells undergoing caspase-dependent apoptosis due to the chemotherapy regimens characterized by gemcitabine and cisplatin. PGE_2_-mediated canonical Wnt signal activation in dormant CSCs was responsible for c-Myc up-regulation and re-entry into the cell cycle [3, 4]. Taken together, the iatrogenic expansion of CSC population is highly likely to ironically cause the “chemotherapy-induced awakening” of dormant CSCs located in MRD with the up-regulation of “wound-response gene signature” in the gene set enrichment analysis (GSEA), which is consistent with the conventional concept of “Cancers as wounds that do not heal [5].”

Wnt signal pathways are mainly classified into β-catenin–dependent canonical pathway and non-canonical pathways. β-catenin plays fundamental roles both in the cell–cell adhesion and in the signal transduction. Whereas β-catenin acts in collaboration with E-cadherin forming adherens junction between epithelial cells, β-catenin dissociated from adaptor protein complex composed of adenomatous polyposis coli (APC), Axin, and glycogen synthase kinase 3-β (GSK3-β) can enter the nucleus and function as a transcriptional factor in association with T cell factor/lymphoid enhancer factor (TCF/LEF). β-catenin phosphorylated by GSK3-β undergoes ubiquitin-proteasome-dependent protein degradation [6]. The expression level of c-Myc is finely regulated by both β-catenin-mediated transcription and E3-ubiquitin-ligase (F-box and WD repeat domain-containing 7; Fbw7)-mediated post-translational degradation [7].

CD44 is one of the CSC markers of various kinds of solid tumors. CD44 is mainly classified into standard and variant isoforms, without or with variable exons, respectively. CD44 standard isoform, which is predominantly expressed in mesenchymal cells, is known to interact with extracellular matrix such as hyaluronic acids. There are several forms of CD44 variant isoforms depending on which variable exons are inserted during the process of the alternative splicing [8]. In particular, CD44 variant 8-10 (CD44v) prevents the accumulation of intracellular reactive oxygen species (ROS) by the stabilization of cystine/glutamate antiporter at the cellular membrane, and subsequently, this CD44v-xCT axis promotes the synthesis of the reduced from of glutathione (GSH) [9]. Given that epithelial splicing regulatory factor 1 (ESRP1) contributes to the alterative splicing to specifically generate CD44v with variable exons 8-10, ESRP1-CD44v-xCT axis is recognized to regulate the redox balance in CSCs. Indeed, ESRP1-CD44v-xCT axis has been demonstrated to enhance the efficiency of breast cancer cells to form metastatic foci in the lungs; CD44v-positive 4T1 murine breast cancer cells are significantly able to metastasize to the lungs as compared with CD44v-negative 4T1 cells [10]. Collectively, ESRP1-CD44v-xCT axis efficiently regulates ROS level in CSCs.

CD44 and c-Myc are target molecules regulated by Wnt/β-catenin signal transduction. It is paradoxical, however, that CD44v and c-Myc tend to show the inversed correlation both in vitro experiments using gastric cancer cell lines and in vivo analyses such as immune-histochemical observations of the xenograft models and human tumor specimens [11]. The detailed molecular machinery underlying the relationship between CD44 and c-Myc remains to be elucidated.

## Presentation of the hypothesis

**Fig. 1:**
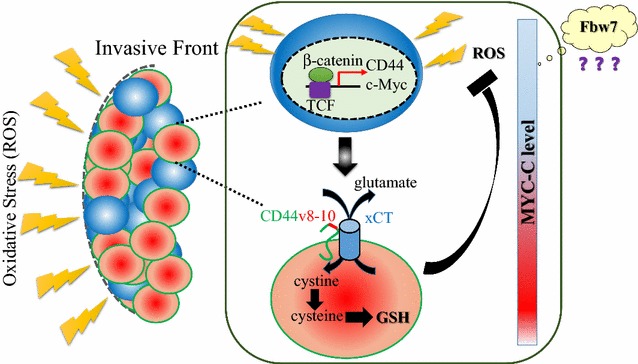
Negative-feedback mechanism of ROS-induced Wnt/β-catenin signal in cancer stem-like cells. The exposure to oxidative stress activates Wnt pathway and up-regulates both CD44 and c-Myc (*blue cells*). CD44v8-10 stabilizes xCT (cystine/glutamate antiporter) and provides CSCs cystine, the rate-limiting substrate of glutathione synthesis. The decreased ROS level due to CD44v-xCT axis influences on ROS-induced Wnt signal in a negative-feedback manner (*red cells*). Both *blue* and *red* cells belong to CSCs, but c-Myc expression level is different each other, which makes the invasive CSCs population quite heterogeneous in terms of cell cycle and proliferation. Although the precise molecular machineries regulated by Fbw7 remain to be unknown, CD44v8-10 and c-Myc tend to exhibit the inversed expression pattern at the invasive front where heterogeneous CSCs are enriched

Mounting evidence has suggested that CSCs are enriched in the heterogeneous manner especially at the invasive/metastatic front of the aggressive tumor. Cancer cells at the invasive area are expected to be composed of both quiescent CSCs exhibiting the expression pattern of CD44v8-10 (high)/Fbw7 (high)/c-Myc (low) and proliferative CSCs exhibiting CD44v8-10 (high)/Fbw7 (low)/c-Myc (high). The major reason why CD44v8-10 and c-Myc tend to show the inversed expression pattern is considered to be due to the negative feedback machinery towards ROS-induced canonical Wnt pathway by CD44v expression (Fig. 1). ESRP1-CD44v-xCT axis has been shown to reduce intracellular ROS level and no longer activates the transcriptional level of c-Myc [11]. Surely, c-Myc is an oncogenic transcriptional factor regulated by not only ROS-induced Wnt/β-catenin signaling but also E-3-ubiquitin-ligase Fbw7. It is still yet to be demonstrated that Fbw7 contributes to the regulation of c-Myc expression to induce heterogeneous CSCs at the invasive/metastatic lesion. It is possible that the heterogeneity of c-Myc stability largely depends on each CSC. Collectively, it is hypothesized that both negative-feedback machinery due to ROS-induced canonical Wnt signal pathway and Fbw7-mediated degradation of oncogenic transcription factor c-Myc are responsible for the heterogeneous distribution of CSCs enriched at the invasive/metastatic foci.

## Testing the hypothesis

To address this hypothesis, it should be noted that both CD44 and c-Myc are regulated by Wnt/β-catenin signal pathway and those molecules are crucial for the maintenance of CSC phenotype at the niche. Increased amount of Wnt ligands promotes c-Myc transcription and accelerates the cell cycle re-entry of G_0_/G_1_-phased dormant CSCs [2, 12]. CD44 has a longer half-life period than c-Myc, one of the oncogenic transcription factors [13]. When Wnt/β-catenin signal pathway is activated due to the increased ROS level, both CD44 and c-Myc are up-regulated at the transcriptional level. However, given the difference in the stabilization as a molecule, c-Myc is expected to be soon down-regulated in CSCs highly expressing CD44v. By striking contrast, CD44 continues to reduce intracellular ROS level by promoting the GSH generation, resulting in the negative feedback machinery of Wnt/β-catenin signaling transduction. On the other hand, there is the heterogeneity in terms of the expression level of c-Myc, which influences on the proliferative potential of each CSC with abundant expression of CD44v. This heterogeneity of c-Myc expression occurs because of the heterogeneous degradation by the ubiquitin-proteasome cascade mainly regulated by Fbw7. So far, it remains to be elusive which Fbw7 isoform mainly contributes to the c-Myc regulation at the invasive/metastatic front. Alternative splicing generates three different protein isoforms of Fbw7 composed of Fbw7α, Fbw7β, and Fbw7γ. Their distribution determines depending on 5′ exon signals that direct the isoforms to distinct subcellular compartments; Fbw7α is nucleoplasmic, Fbw7β is cytoplasmic, and Fbw7γ is nucleolar, respectively. Furthermore, there are no reliable antibodies available for Fbw7. Importantly, it would be difficult to identify the heterogeneity of CSC population at the invasive front derived from the biopsy or surgical specimen. CSCs are critically influenced by the microenvironment, which is why the complexity of heterogeneous CSCs at the invasive/metastatic lesion would exhibit the discrepancy as compared with the bona fide behavior of invasive CSC population.

## Implications of the hypothesis

ROS is a double-edged sword for cancer cells. While the excessive level of ROS can induce apoptotic or necrotic cell death, the appropriate range of ROS level triggers cellular proliferation by influencing on several survival signal transductions characterized by canonical Wnt/β-catenin pathway. Upon exposure to redox stress, nucleoredoxin (NRX) associated with redox-sensitive Wnt adaptor protein inhibits the ubiquitin ligase activity of GSK3β and leads to β-catenin stabilization. β-Catenin spared from phosphorylation by GSK3β translocates into the nucleus and binds to TCF/LEF promoter, thereby enhancing the transcription of *Axin*-*2*, *CD44*, *c*-*Myc*, *CCND1*, and *β*-*catenin* itself [14]. Thus, ROS can be either beneficial or harmful for cancer cells in the microenvironment.

Furthermore, the detailed molecular mechanism underlying the inversed expression pattern between CSC marker CD44v and oncogenic transcription factor c-Myc should be elucidated to better understand how aggressively invasive/metastatic CSCs simultaneously regulate cell cycle and ROS level. c-Myc drives the cellular proliferation and causes metabolic reprogramming of tumor cells. It has already been shown that Ki-67, the proliferative marker, exhibits the reversed expression pattern as compared with CD44v [11]. From the perspective of tumor metabolism, c-Myc has been revealed to stimulate the expression of both Na^+^-dependent amino acid transporter ASCT2 and glutaminases (GLS) which is an essential enzyme for glutaminolysis. c-Myc promotes glutamine uptake via ASCT2 transporter and glutamine catabolism mediated by GLS mainly in the mitochondria. This excessive dependency on glutamine catabolism in cancer cells is referred to as “glutamine addiction [15] ”. It is likely that heterogeneous CSC population composed of dormant CSCs with CD44v8-10 (high)/Fbw7 (high)/c-Myc (low) and proliferative CSCs with CD44v8-10 (high)/Fbw7 (low)/c-Myc (high) exhibit the metabolic symbiosis at the invasive/metastatic front.

While CD44v brings about the negative feedback loop against ROS-induced canonical Wnt signal activation, c-Myc would be easily down-regulated as compared with CD44 in terms of the difference in the half-life period. That is why CD44, especially CD44v8-10, and c-Myc tend to show the inversed expression pattern each other. In addition, the heterogeneous amount and activity of Fbw7 ubiquitin ligase makes the heterogeneity of oncogenic c-Myc expression. This heterogeneous c-Myc expression at the invasive/metastatic lesion represents the diversity in the proliferative capacity of CSC population with robust CD44v expression. When researchers demonstrate the regulation machinery underlying the heterogeneous CSCs at the invasive/metastatic edge, the therapeutic strategy against tumor invasion and metastasis by targeting CSCs would be much more validated. As mentioned above [3], the chemotherapy can stimulate dormant CSCs and induce the therapeutic resistance to the conventionally effective drugs. Collectively, the heterogeneous CSCs at the invasive/metastatic edge is expected to reveal the dynamic evolution with the selective pressure of anti-cancer treatments.

Last but certainly not least, the novel molecular targeting therapies against aggressive CSCs would be more warranted. Sulfasalazine, the conventional drug for the treatment of rheumatoid arthritis and ulcerative colitis, has been revealed to inhibit the function of xCT transporter [9, 10]. This is the typical example of the drug re-positioning, finding out the novel anti-tumor effect of conventionally used agents. As compared with novel molecular-targeting drugs, conventionally-used agents are pharmacologically safe and beneficial for medical economy. Indeed, sulfasalazine has been shown to reduce the proliferation of CD44v-highly expressing cancer cells in the clinical trials on advanced gastric and pulmonary tumors [2, 15]. CSCs enriched at the invasive/metastatic front with enhanced level of ROS are likely to disappear, when ERSP1-CD44v-xCT axis is disrupted by sulfasalazine treatment. Given that c-Myc drives the cell division and promotes the glutamine metabolism [15], the molecular targeting drug for this oncogenic transcriptional factor would show the synergistic therapeutic effect against CSCs at the invasive/metastatic front with xCT inhibitor.

## Abbreviations

APC: adenomatosis polyposis coli
CD44v: CD44 variant 8-10
CSCs: cancer stem-like cells
ESRP1: epithelial splicing regulatory factor 1
Fbw7: F-box and WD repeat domain-containing 7
GLS: glutaminases
GSEA: gene set enrichment analysis
GSH: reduced form of glutathione
GSK3-β: glycogen synthase kinase 3-β
MRD: minimal residual disease
NRX: nucleoredoxin
PGE_2_: prostaglandin E_2_
ROS: reactive oxygen species
TCF/LEF: T cell factor/lymphoid enhancer factor
